# Supplementary material for: An adaptive behavioral control motif mediated by cortical axo-axonic inhibition
Source: Nat Neurosci. 2023 Jul 20;26(8):1379–93. doi: 10.1038/s41593-023-01380-x (PMC10400431; doi:10.1038/s41593-023-01380-x)
Supplement: Supplementary file 1 — Supplementary Tables 1 and 2. [file 41593_2023_1380_MOESM1_ESM.pdf]

nature neuroscience

Article

https://doi.org/10.1038/s41593-023-01380-x

An adaptive behavioral control motif mediated by cortical axo-axonic inhibition

In the format provided by the authors and unedited

Supplementary Table 1. Statistical results:

Figure	Description	Test	Statistics	p-value	Number of samples
Fig. 1c	Average movement speed	Two-way repeated measures ANOVA	F_{group} (1,6) = 3.85	P = 0.072	n = 27 for the experimental group; n = 14 for the control group.
Fig. 1d	Average movement acceleration	Two-way repeated measures ANOVA	F_{group} (1,6) = 1.54	P = 0.24	n = 27 for the experimental group; n = 14 for the control group.
Fig. 1e	Average number of successes obtained in training sessions	Two-way repeated measures ANOVA	F_{group} (1,6) = 26.97	P = 1.73×10^{-4}	n = 27 for the experimental group; n = 14 for the control group.
Fig. 1f	Average latency to reward	Two-way repeated measures ANOVA	F_{group} (1,6) = 7.43	P = 0.017	n = 27 for the experimental group; n = 14 for the control group.
Fig. 1g	Average goal proximity	Two sample t-test	t = -5.19 for Exp;<br>t = 1.15 for Control	P = 1.03×10^{-6} for Exp;<br>P = 0.26 for Control	n = 27 for the experimental group; n = 14 for the control group.
Fgi. 1h	Movement accuracy	Two sample t-test	t = -7.04 for Exp;<br>t = 1.74 for Control	P = 2.01×10^{-10} for Exp;<br>P = 0.09 for Control	n = 27 for the experimental group; n = 14 for the control group.
Fig. 1j	Comparison of cumulative turning angle between Exp and Control	Two sample t-test	t = -5.84 for Exp;<br>t = 0.019 for Control	P = 5.76×10^{-8} for Exp;<br>P = 0.98 for Control	n = 27 for the experimental group;<br>n = 14 for the control group.
Fig. 2d	Comparison of population response precisions between early and late sessions	Two-tailed paired t-test	t = -4.98 for Exp;<br>t= 2.56 for Control	P = 0.0042 for Exp;<br>P = 0.063 for Control	n = 6 mice for Exp;<br>n = 5 mice for Control
Fig. 2f	Changes of the percentage of active cells from early session and late session	Two-tailed Student's t-test	t = 5.149 for Exp;<br>t= 0.106 for Control	P = 0.0036 for Exp;<br>P = 0.920 for Control	n = 6 mice for Exp; n = 5 mice for Control
Fig. 3a	Behavioral	One-way repeated	$F_{session}$ (2,14) =	P = 8.69×10^{-6} for	n = 8 mice for PV-

	performance of the number of successes with training and photo-inhibition	measures ANOVA	30.0 for PV-NpHR; $F_{session}$ (2,8) = 0.67 for SOM-NpHR	PV-NpHR;<br>P = 0.67 for SOM-NpHR	NpHR;<br>n = 5 mice for SOM-NpHR
Fig. 3h	Population fraction of neuronal pairs with positive and negative correlation	Chi-square test	χ^2 = 235.8	P = 0	n = 31660 pairs from 6 mice for PV-TeTxLC;<br>n = 24061 pairs from 7 mice for PV control
Fig. 4f	Population fraction of neuronal pairs with positive and negative correlation	Chi-square test	χ^2 = 1.21	P = 0.27	n = 14082 pairs from 5 mice for ChC-TeTxLC;<br>n = 57956 pairs from 4 mice for ChC control
Fig. 5c	The average number of successes of ChC-hM4Di mice	One-way repeated measures ANOVA for Sessions 7, 8, and 9 followed by Fisher post hoc tests	$F_{session}$ (2,16) = 31.1	P = 3.09×10^{-6}; Fisher multiple comparisons tests (Session 7 vs. CNO, P = 2.40×10^{-6}; Session CNO vs. Saline, P = 7.58×10^{-6}; Session 7 vs. Saline, P = 0.53).	n = 9 mice
Fig. 5d	Comparison of performance between conditions	One-way ANOVA followed by Fisher post hoc tests	F_{group} (3,26) = 9.50	P = 2.06×10^{-4}; Fisher multiple comparisons tests (Control-CNO vs. Control- Saline, P = 0.20; hM4Di-Saline vs. Control-Saline, P = 0.78; hM4Di-Saline vs. Control-CNO, P = 0.097; hM4Di-CNO vs. Control-Saline, P = 0.0016; hM4Di-CNO vs. Control-CNO, P = 3.79×10^{-5}; hM4Di-CNO vs. hM4Di-Saline, P = 0.0013).	n = 6 mice for ChC control with saline or CNO; n = 9 mice for ChC-hM4Di with saline or CNO
Fig. 5f	Cumulative turning angle of ChC-hM4Di between conditions	Friedman test<br>for Sessions 7, 8, and 9 followed by Dunn post hoc tests	$\chi^2(2)$ = 13 for PV-NpHR;<br>$\chi^2(2)$ = 2.8 for SOM-NpHR	P = 0.0015 for PV-NpHR;<br>P = 0.25 for SOM-NpHR	n = 8 mice for PV-NpHR;<br>n = 5 mice for SOM-NpHR
Fig. 5i	Coactivity percentage of	Friedman test	$\chi^2(2)$ = 9.33 for locomotion;	P = 0.0094 for locomotion;	n = 6 ChC-hM4Di mice

	neurons during periods of locomotion and rest		$\chi^2(2) = 0.33$ for rest	P = 0.85 for rest	
Fig. 5l	Comparison of population response precisions	One-way repeated measures ANOVA	$F_{session,}$ (2,10) = 6.39	P = 0.016; Fisher multiple comparisons tests (Session 7 vs. CNO, P = 0.0068; Session CNO vs. Saline, P = 0.023; Session 7 vs. Saline, P = 0.486).	n = 6 ChC-hM4Di mice
Fig. 5n	Changes in the percentage of active cells across conditions.	One-way repeated measures ANOVA with Greenhouse-Geisser correction for Sessions 7, 8, and 9 followed by Fisher post hoc tests	$F_{session}$ (1.11,5.53) = 7.26	P = 0.037; Fisher multiple comparisons tests (Session 7 vs. CNO, P = 0.006; Session CNO vs. Saline, P = 0.011; Session 7 vs. Saline, P = 0.372).	n = 6 ChC-hM4Di mice
Fig. 6d	Changes in correlation between a pair of ChCs	Two-tailed Wilcoxon signed-rank test	Z = 11.92	P = 0	n = 189 pairs from 35 cells from 3 mice
Fig. 6g	Cell-to-cell Pearson's correlation of ChC-PyN pairs in during locomotion and rest epochs	Two-sample Kolmogorov-Smirnov test	D = 0.076 for Session 1; D = 0.28 for Session 7	$P = 2.12 \times 10^{-5}$ for Session 1; $P = 4.049 \times 10^{-61}$ for Session 7	n = 1957 pairs in Session 1; n = 1723 pairs in Session 7 from 4 ChC-hM4Di mice
Fig. 7e	Average movement speed	Two-way repeated measures ANOVA	F_{group} (1,6) = 1.16	P = 0.32	n = 9 for ChC-TeTxLC group; n = 8 for ChC control group
Fig. 7f	Average movement acceleration	Two-way repeated measures ANOVA	F_{group} (1,6) = 3.22	P = 0.12	n = 9 for ChC-TeTxLC group; n = 8 for ChC control group
Fig. 7g	Average number of successes obtained in training sessions	Two-way repeated measures ANOVA	F_{group} (1,6) = 8.54	P = 0.011	n = 9 for ChC-TeTxLC group; n = 8 for ChC control group
Fig. 7h	Average latency to reward	Two-way repeated measures ANOVA	F_{group} (1,6) = 7.81	P = 0.0136	n = 9 for ChC-TeTxLC group; n = 8 for ChC control group
Fig. 7i	Average goal proximity	Two sample t-test	t = 1.38 for ChC-TeTxLC; t = -2.52 for	P = 0.177 for ChC-TeTxLC; P = 0.017 for ChC control	n = 9 for ChC-TeTxLC group; n = 8 for ChC control group

			ChC control		
Fig. 7j	Movement accuracy	Two sample t-test	t = -1.05 for ChC-TeTxLC; t = -3.8 for ChC control	P = 0.3 for ChC-TeTxLC; P = 6.67 × 10^{-4} for ChC control	n = 9 for ChC-TeTxLC group; n = 8 for ChC control group
Fig. 7l	Comparison of cumulative turning angle between ChC-TeTxLC and ChC control	Two sample t-test	t = -1.39 for ChC-TeTxLC; t = -3.21 for ChC control	P = 0.175 for ChC-TeTxLC; P = 3.51 × 10-3 for ChC control	n = 9 for ChC-TeTxLC group; n = 8 for ChC control group
Fig. 7o	Changes of the percentage of active cells from session 1 to session 7	Two-tailed Student's t-test	t = 0.56 for ChC-TeTxLC; t = -17.97 for ChC control	P = 0.606 for ChC-TeTxLC; P = 3.76 × 10^{-4} for ChC control	n = 5 for ChC-TeTxLC group; n = 4 for ChC control group
Fig. 8i	Pre-SSE vs. Post-SSE	Pearson's Linear Correlation Coefficient	rho = 0.7616 / R^2 = 0.58 (control)<br>rho = 0.7033 / R^2 = 0.495 (experimental)	P = 0 (control)<br>P = 4.072 x 10^{-244} (experimental)	n = 3116 for the control group;<br>n = 1634 for the experimental group.
Fig. 8j upper panel	Pre-SSEs by Depth from L1/2	Two-tailed Wilcoxon-Mann-Whitney	U =<br>6<br>10<br>15<br>13<br>9<br>6<br>10<br>4<br>9<br>-11	P =<br>0.1255 0.4286<br>1.0000<br>0.7922<br>0.3290<br>0.1255<br>0.4286<br>0.6095<br>0.3290<br>0.1429	n = 5 for the experimental group;<br>n = 6 for the control group.
Fig. 8j lower panel	Post-SSEs by Depth from L1/2	Two-tailed Wilcoxon-Mann-Whitney	U =<br>12<br>9<br>15<br>13<br>14<br>10<br>12<br>1<br>6<br>-11	P =<br>0.6623<br>0.3290<br>1.0000<br>0.7922<br>0.9307<br>0.4286<br>0.6623<br>0.2571<br>0.1255<br>0.2143	n = 5 for the experimental group;<br>n = 6 for the control group.
Fig. 8k	CDF of Pre-SSE	Two-sample Kolmogorov-Smirnov	D = 0.0698	P = 5.311x10^{-5}	n = 5 for the experimental group;<br>n = 6 for the control group.
Fig. 8l	CDF of Post-SSE	Two-sample Kolmogorov-Smirnov	D = 0.1084	P = 1.860x10^{-11}	n = 5 for the experimental group;

					n = 6 for the control group.
Fig. 8q	Proportional change of Pre-SSE in bootstrapping test	Two-tailed Wilcoxon-Mann-Whitney	U = 0 for all bins	$P = 1.827x10^{-4}$	n = 10 for the experimental group and for the control group.
Fig. 8r	Proportional change of Post-SSE in bootstrapping test	Two-tailed Wilcoxon-Mann-Whitney	U = 0 for all bins	$P = 1.827x10^{-4}$	n = 10 for the experimental group and for the control group.
EDF. 1c	Average movement speed of CaMKII-TeTxLC	Two-way repeated measures ANOVA	F_{group} (1,6) = 0.45	P = 0.53	n = 27 for the experimental group; n = 7 for CaMKII-TeTxLC
EDF. 1d	Average movement acceleration of CaMKII-TeTxLC	Two-way repeated measures ANOVA	F_{group} (1,6) = 19.59	P = 0.0044	n = 27 for the experimental group; n = 7 for CaMKII-TeTxLC
EDF. 1e	Average number of successes obtained in training sessions	Two-way repeated measures ANOVA	F_{group} (1,6) = 24.11	P = 0.0027	n = 27 for the experimental group; n = 7 for CaMKII-TeTxLC
EDF. 1f	Average latency to reward of CaMKII-TeTxLC	Two-way repeated measures ANOVA	F_{group} (1,6) = 21.75	P = 0.0035	n = 27 for the experimental group; n = 7 for CaMKII-TeTxLC
EDF. 1g	Average goal proximity of CaMKII-TeTxLC	Two sample t-test	t = -5.19 for Exp;<br>t = -0.06 for CaMKII-TeTxLC	$P = 1.03 \times 10^{-6}$ for Exp;<br>P = 0.95 for CaMKII-TeTxLC	n = 27 for the experimental group;<br>n = 7 for CaMKII-TeTxLC
EDF. 1h	Movement accuracy of CaMKII-TeTxLC	Two sample t-test	t = -7.04 for Exp;<br>t = -1.54 for CaMKII-TeTxLC	$P = 2.01 \times 10^{-10}$ for Exp;<br>P = 0.135 for CaMKII-TeTxLC	n = 27 for the experimental group;<br>n = 7 for CaMKII-TeTxLC
EDF. 1j	Comparison of cumulative turning angle between Exp and CaMKII-TeTxLC	Two sample t-test	t = -5.84 for Exp;<br>t = -2.05 for CaMKII-TeTxLC	$P = 5.76 \times 10^{-8}$ for Exp;<br>P = 0.050 for CaMKII-TeTxLC	n = 27 for the experimental group;<br>n = 7 for CaMKII-TeTxLC

EDF. 3i	Changes of cumulative distribution of similarity index between PoV and MD with learning	Two-sample Kolmogorov-Smirnov test	D = 0.0495 for Exp;<br>D = 0.027 for Control	P = 0.0003 for Exp;<br>P = 0.12 for Control	n = 6 mice for the experimental group;<br>n = 5 mice for the control group
EDF. 3k	Changes of the cumulative distribution of similarity index between active neurons' PD and MD with learning	Two-sample Kolmogorov-Smirnov test	D = 0.0533 for Exp;<br>D = 0.0584 for Control	P = 2.68×10^{-10} for Exp;<br>P = 2.61×10^{-10} for Control	n = 6 mice for the experimental group;<br>n = 5 mice for the control group
EDF. 4b	Average speed of PV-NpHR and SOM-NpHR	One-way repeated measures ANOVA with Greenhouse-Geisser correction for Sessions 7, 8, and 9 followed by Fisher post hoc tests	$F_{session}$ (1.13,7.92) = 10.16 for PV-NpHR; $F_{session}$ (1.37, 5.46)= 1.08 for SOM-NpHR	P = 0.0019 for PV-NpHR;<br>P = 0.37 for SOM-NpHR	n = 8 mice for PV-NpHR;<br>n = 5 mice for SOM-NpHR
EDF. 4c	Average acceleration of PV-NpHR and SOM-NpHR	One-way repeated measures ANOVA for Sessions 7, 8, and 9 followed by Fisher post hoc tests	$F_{session}$ (2,14) = 0.45 for PV-NpHR; $F_{session}$ (2,8) = 3.69 for SOM-NpHR	P = 0.65 for PV-NpHR;<br>P = 0.07 for SOM-NpHR	n = 8 mice for PV-NpHR;<br>n = 5 mice for SOM-NpHR
EDF. 4d	Average latency to reward of PV-NpHR and SOM-NpHR	One-way repeated measures ANOVA with Greenhouse-Geisser correction for Sessions 7, 8, and 9 followed by Fisher post hoc tests	$F_{session}$ (1.12,7.81) = 9.55 for PV-NpHR; $F_{session}$ (1.36,5.43) = 0.094 for SOM-NpHR	P = 0.014 for PV-NpHR;<br>P = 0.84 for SOM-NpHR	n = 8 mice for PV-NpHR;<br>n = 5 mice for SOM-NpHR
EDF. 4e	Average goal proximity of PV-NpHR and SOM-NpHR	One-way repeated measures ANOVA with Greenhouse-Geisser correction for Sessions 7, 8, and 9	$F_{session}$ (1.45,10.15) = 40.67 for PV-NpHR; $F_{session}$ (1.26,5.04) = 1.57 for SOM-NpHR	P = 3.05×10^{-5} for PV-NpHR; P = 0.28 for SOM-NpHR	n = 8 mice for PV-NpHR;<br>n = 5 mice for SOM-NpHR
EDF. 4f	Movement accuracy of PV-NpHR and SOM-NpHR	One-way repeated measures ANOVA with Greenhouse-Geisser correction for Sessions 7, 8, and 9	$F_{session}$ (2,14)= 13.9 for PV-NpHR; $F_{session}$ (1.19,4.77) = 3.06 for SOM-NpHR	P = 4.72×10^{-4} for PV-NpHR; P = 0.14 for SOM-NpHR	n = 8 mice for PV-NpHR;<br>n = 5 mice for SOM-NpHR
EDF. 4h	Cumulative turning angle of PV-NpHR and SOM-	Friedman test for Sessions 7, 8, and 9 followed by Dunn post hoc tests	$\chi^2(2)$ = 13 for PV-NpHR;<br>$\chi^2(2)$ = 2.8 for SOM-NpHR	P = 0.0015 for PV-NpHR;<br>P = 0.25 for SOM-NpHR	n = 8 mice for PV-NpHR;<br>n = 5 mice for SOM-NpHR

	NpHR				
EDF. 6c	Density of cells expressing TeTxLC	One-way ANOVA	F_{group} (2,20) = 0.62	P = 0.547	n = 7 mice for sparse PV-TeTxLC;<br>n= 5 mice for expression of ChC-TeTxLC;<br>n= 11 mice for expression of FLEX-tdTomato in Vipr2-Cre mice
EDF. 6e	The number of successes for sparse PV-TeTxLC and PV control mice	Two-way repeated measures ANOVA	F_{group} (1,6) = 0.050	P = 0.831	n = 7 mice for sparse PV-TeTxLC;<br>n = 7 mice for PV control
EDF. 6f	The latency to reward for sparse PV-TeTxLC and PV control mice	Two-way repeated measures ANOVA	F_{group} (1,6) = 0.346	P = 0.578	n = 7 mice for sparse PV-TeTxLC;<br>n = 7 mice for PV control
EDF. 8g	Pre-SSE in bootstrapping test between groups	Two-tailed Wilcoxon-Mann-Whitney	Check Table 2	Presented in the plot	n = 3000 for random sampling in the null group (no difference) and each conditional group; n = 10 for repeated sampling
EDF. 8i	Post-SSE in bootstrapping test between groups	Two-tailed Wilcoxon-Mann-Whitney	Check Table 2	Presented in the plot	n = 3000 for random sampling in the null group (no difference) and each conditional group; n = 10 for repeated sampling

Supplementary Table 2. U-stats for EDF 8g-i

	C vs C							
Pre-SSE	0.5	1	1.5	2	2.5	3	3.5	4
1	4E+06	4E+06	4E+06	4E+06	4E+06	4E+06	4E+06	4E+06
2	4E+06	4E+06	4E+06	4E+06	4E+06	4E+06	4E+06	4E+06
3	4E+06	4E+06	4E+06	4E+06	4E+06	4E+06	4E+06	4E+06
4	4E+06	4E+06	4E+06	4E+06	4E+06	4E+06	4E+06	4E+06
5	4E+06	4E+06	4E+06	4E+06	4E+06	4E+06	4E+06	4E+06
6	4E+06	4E+06	4E+06	4E+06	4E+06	4E+06	4E+06	4E+06
7	4E+06	4E+06	4E+06	4E+06	4E+06	4E+06	4E+06	4E+06
8	4E+06	4E+06	4E+06	4E+06	4E+06	4E+06	4E+06	4E+06
9	4E+06	4E+06	4E+06	4E+06	4E+06	4E+06	4E+06	4E+06
10	4E+06	4E+06	4E+06	4E+06	4E+06	4E+06	4E+06	4E+06

	C vs C									
Post-SSI	0.1	0.2	0.3	0.4	0.5	0.6	0.7	0.8	0.9	1
1	4E+06	4E+06	4E+06	5E+06	4E+06	4E+06	4E+06	4E+06	4E+06	4E+06
2	4E+06	4E+06	4E+06	4E+06	4E+06	4E+06	4E+06	4E+06	4E+06	4E+06
3	4E+06	4E+06	4E+06	4E+06	4E+06	4E+06	4E+06	4E+06	4E+06	4E+06
4	4E+06	4E+06	4E+06	4E+06	4E+06	4E+06	4E+06	4E+06	4E+06	4E+06
5	4E+06	4E+06	4E+06	4E+06	4E+06	4E+06	5E+06	4E+06	4E+06	4E+06
6	4E+06	4E+06	4E+06	4E+06	4E+06	4E+06	4E+06	4E+06	4E+06	4E+06
7	4E+06	4E+06	4E+06	4E+06	4E+06	4E+06	4E+06	4E+06	4E+06	4E+06
8	4E+06	4E+06	4E+06	4E+06	4E+06	4E+06	4E+06	4E+06	4E+06	4E+06
9	5E+06	4E+06	4E+06	4E+06	4E+06	4E+06	4E+06	4E+06	4E+06	4E+06
10	4E+06	4E+06	4E+06	4E+06	4E+06	4E+06	4E+06	4E+06	4E+06	4E+06

	E vs E							
Pre-SSE	0.5	1	1.5	2	2.5	3	3.5	4
1	4E+06	4E+06	4E+06	4E+06	4E+06	4E+06	4E+06	4E+06
2	4E+06	4E+06	4E+06	4E+06	4E+06	4E+06	4E+06	4E+06
3	4E+06	4E+06	4E+06	4E+06	4E+06	4E+06	4E+06	4E+06
4	4E+06	4E+06	4E+06	4E+06	4E+06	4E+06	4E+06	4E+06
5	4E+06	4E+06	4E+06	4E+06	4E+06	4E+06	4E+06	4E+06
6	4E+06	4E+06	4E+06	4E+06	4E+06	4E+06	4E+06	4E+06
7	4E+06	4E+06	4E+06	4E+06	4E+06	4E+06	4E+06	4E+06
8	4E+06	4E+06	4E+06	4E+06	4E+06	4E+06	4E+06	4E+06
9	4E+06	4E+06	4E+06	4E+06	4E+06	4E+06	4E+06	4E+06
10	4E+06	4E+06	4E+06	4E+06	4E+06	4E+06	4E+06	4E+06

	E vs E									
Post-SSI	0.1	0.2	0.3	0.4	0.5	0.6	0.7	0.8	0.9	1
1	4E+06	4E+06	4E+06	4E+06	4E+06	4E+06	4E+06	4E+06	4E+06	4E+06
2	4E+06	4E+06	4E+06	4E+06	4E+06	4E+06	4E+06	4E+06	4E+06	4E+06
3	4E+06	4E+06	4E+06	4E+06	4E+06	4E+06	4E+06	4E+06	4E+06	4E+06
4	4E+06	4E+06	4E+06	4E+06	4E+06	4E+06	4E+06	4E+06	4E+06	4E+06
5	4E+06	4E+06	4E+06	4E+06	4E+06	4E+06	4E+06	4E+06	4E+06	4E+06
6	4E+06	5E+06	4E+06	4E+06	5E+06	4E+06	4E+06	5E+06	4E+06	4E+06
7	4E+06	4E+06	4E+06	4E+06	4E+06	4E+06	4E+06	4E+06	4E+06	4E+06
8	4E+06	4E+06	4E+06	4E+06	4E+06	4E+06	4E+06	4E+06	4E+06	4E+06
9	4E+06	4E+06	4E+06	4E+06	4E+06	4E+06	4E+06	4E+06	4E+06	4E+06
10	4E+06	4E+06	4E+06	4E+06	4E+06	4E+06	5E+06	4E+06	4E+06	4E+06

	U vs U							
Pre-SSE	0.5	1	1.5	2	2.5	3	3.5	4
1	4E+06	4E+06	4E+06	4E+06	4E+06	4E+06	4E+06	4E+06
2	4E+06	4E+06	4E+06	4E+06	4E+06	4E+06	4E+06	4E+06
3	4E+06	4E+06	4E+06	4E+06	4E+06	4E+06	4E+06	4E+06
4	5E+06	4E+06	4E+06	4E+06	4E+06	5E+06	4E+06	4E+06
5	4E+06	4E+06	4E+06	4E+06	4E+06	4E+06	4E+06	4E+06
6	4E+06	4E+06	4E+06	4E+06	4E+06	4E+06	4E+06	4E+06
7	4E+06	4E+06	4E+06	4E+06	4E+06	4E+06	4E+06	4E+06
8	4E+06	4E+06	4E+06	4E+06	4E+06	4E+06	4E+06	4E+06
9	4E+06	4E+06	4E+06	4E+06	4E+06	4E+06	4E+06	4E+06
10	4E+06	4E+06	4E+06	4E+06	4E+06	4E+06	4E+06	4E+06

	U vs U									
Post-SSI	0.1	0.2	0.3	0.4	0.5	0.6	0.7	0.8	0.9	1
1	4E+06	4E+06	4E+06	4E+06	4E+06	4E+06	4E+06	4E+06	4E+06	4E+06
2	4E+06	4E+06	4E+06	4E+06	4E+06	4E+06	4E+06	4E+06	4E+06	4E+06
3	4E+06	4E+06	4E+06	4E+06	4E+06	4E+06	4E+06	4E+06	4E+06	4E+06
4	4E+06	4E+06	4E+06	4E+06	4E+06	4E+06	4E+06	4E+06	4E+06	4E+06
5	4E+06	4E+06	4E+06	4E+06	4E+06	4E+06	4E+06	4E+06	4E+06	4E+06
6	4E+06	4E+06	4E+06	4E+06	4E+06	4E+06	4E+06	4E+06	4E+06	4E+06
7	4E+06	4E+06	4E+06	4E+06	4E+06	4E+06	4E+06	4E+06	4E+06	4E+06
8	4E+06	4E+06	4E+06	4E+06	4E+06	4E+06	4E+06	4E+06	4E+06	4E+06
9	4E+06	4E+06	5E+06	4E+06	5E+06	4E+06	4E+06	4E+06	4E+06	4E+06
10	4E+06	4E+06	4E+06	4E+06	4E+06	4E+06	4E+06	4E+06	4E+06	4E+06

	E vs C							
Pre-SSE	0.5	1	1.5	2	2.5	3	3.5	4
1	4E+06	2E+06	4E+06	4E+06	4E+06	3E+06	4E+06	4E+06
2	4E+06	2E+06	4E+06	4E+06	4E+06	3E+06	4E+06	4E+06
3	4E+06	2E+06	4E+06	3E+06	4E+06	3E+06	4E+06	4E+06
4	4E+06	2E+06	4E+06	3E+06	4E+06	3E+06	4E+06	4E+06
5	4E+06	2E+06	3E+06	4E+06	4E+06	3E+06	4E+06	4E+06
6	4E+06	2E+06	4E+06	4E+06	4E+06	3E+06	4E+06	4E+06
7	4E+06	2E+06	4E+06	4E+06	4E+06	3E+06	4E+06	4E+06
8	4E+06	2E+06	4E+06	4E+06	4E+06	3E+06	4E+06	4E+06
9	4E+06	2E+06	4E+06	4E+06	4E+06	3E+06	4E+06	4E+06
10	4E+06	2E+06	4E+06	4E+06	4E+06	3E+06	4E+06	4E+06

	E vs C									
Post-SSI	0.1	0.2	0.3	0.4	0.5	0.6	0.7	0.8	0.9	1
1	2E+06	2E+06	2E+06	2E+06	1E+06	4E+06	3E+06	4E+06	3E+06	2E+06
2	2E+06	1E+06	2E+06	2E+06	2E+06	4E+06	3E+06	4E+06	3E+06	2E+06
3	2E+06	2E+06	2E+06	2E+06	1E+06	4E+06	3E+06	4E+06	3E+06	2E+06
4	2E+06	1E+06	2E+06	2E+06	1E+06	4E+06	3E+06	4E+06	3E+06	2E+06
5	2E+06	1E+06	2E+06	2E+06	2E+06	4E+06	3E+06	4E+06	3E+06	2E+06
6	2E+06	1E+06	2E+06	2E+06	2E+06	4E+06	3E+06	4E+06	3E+06	2E+06
7	2E+06	1E+06	2E+06	2E+06	2E+06	4E+06	3E+06	4E+06	3E+06	2E+06
8	2E+06	1E+06	2E+06	2E+06	1E+06	4E+06	3E+06	4E+06	3E+06	2E+06
9	2E+06	2E+06	2E+06	2E+06	1E+06	4E+06	3E+06	4E+06	3E+06	2E+06
10	2E+06	1E+06	2E+06	2E+06	1E+06	4E+06	3E+06	4E+06	3E+06	2E+06
