# Supplementary material for: An adaptive behavioral control motif mediated by cortical axo-axonic inhibition
Source: Nat Neurosci. 2023 Jul 20;26(8):1379–93. doi: 10.1038/s41593-023-01380-x (PMC10400431; doi:10.1038/s41593-023-01380-x)
Supplement: Supplementary file 2 — Reporting Summary [file 41593_2023_1380_MOESM2_ESM.pdf]

Corresponding author(s): Hyung-Bae Kwon
Last updated by author(s): Jun 5, 2023

Reporting Summary

Nature Portfolio wishes to improve the reproducibility of the work that we publish. This form provides structure for consistency and transparency in reporting. For further information on Nature Portfolio policies, see our Editorial Policies and the Editorial Policy Checklist.

Statistics

For all statistical analyses, confirm that the following items are present in the figure legend, table legend, main text, or Methods section.

n/a	Confirmed	
☐	☒	The exact sample size (*n*) for each experimental group/condition, given as a discrete number and unit of measurement
☐	☒	A statement on whether measurements were taken from distinct samples or whether the same sample was measured repeatedly
☐	☒	The statistical test(s) used AND whether they are one- or two-sided<br>*Only common tests should be described solely by name; describe more complex techniques in the Methods section.*
☐	☒	A description of all covariates tested
☐	☒	A description of any assumptions or corrections, such as tests of normality and adjustment for multiple comparisons
☐	☒	A full description of the statistical parameters including central tendency (e.g. means) or other basic estimates (e.g. regression coefficient) AND variation (e.g. standard deviation) or associated estimates of uncertainty (e.g. confidence intervals)
☐	☒	For null hypothesis testing, the test statistic (e.g. *F*, *t*, *r*) with confidence intervals, effect sizes, degrees of freedom and *P* value noted<br>*Give P values as exact values whenever suitable.*
☐	☒	For Bayesian analysis, information on the choice of priors and Markov chain Monte Carlo settings
☒	☐	For hierarchical and complex designs, identification of the appropriate level for tests and full reporting of outcomes
☐	☒	Estimates of effect sizes (e.g. Cohen's *d*, Pearson's *r*), indicating how they were calculated

Our web collection on statistics for biologists contains articles on many of the points above.

Software and code

Policy information about availability of computer code

Data collection	Licenses for MATLAB2017a/2018a/2019a/2021a, Origin 2019b, and Excel are commercially available, and Fiji software can be freely downloaded at https://fiji.sc/.
Data analysis	All quantification methods used in the custom scripts are described in Method Details.<br>The code for semi-automated ROI selection can be found at https://github.com/fitzlab/CellMagicWand. All custom MATLAB codes and the data analyzed for this study are available at https://github.com/KanghoonJ/Jung_NatNeuro_2023. Source data are provided with this paper. . Further requests for data used in this study can be directed to the corresponding author (hkwon29@jhmi.edu).

For manuscripts utilizing custom algorithms or software that are central to the research but not yet described in published literature, software must be made available to editors and reviewers. We strongly encourage code deposition in a community repository (e.g. GitHub). See the Nature Portfolio guidelines for submitting code & software for further information.

Data

Policy information about availability of data

All manuscripts must include a data availability statement. This statement should provide the following information, where applicable:

- Accession codes, unique identifiers, or web links for publicly available datasets
- A description of any restrictions on data availability
- For clinical datasets or third party data, please ensure that the statement adheres to our policy

The data analyzed for this study are available at https://github.com/KanghoonJ/Jung_NatNeuro_2023. Source data are provided with this paper. Additional data that supports the findings of this study are available from the corresponding author upon reasonable request. Further requests for data used in this study can be directed to the corresponding author (hkwon29@jhmi.edu).

Human research participants

Policy information about studies involving human research participants and Sex and Gender in Research.

Reporting on sex and gender	N/A
Population characteristics	N/A
Recruitment	N/A
Ethics oversight	N/A

Note that full information on the approval of the study protocol must also be provided in the manuscript.

Field-specific reporting

Please select the one below that is the best fit for your research. If you are not sure, read the appropriate sections before making your selection.

☒ Life sciences ☐ Behavioural & social sciences ☐ Ecological, evolutionary & environmental sciences

For a reference copy of the document with all sections, see nature.com/documents/nr-reporting-summary-flat.pdf

Life sciences study design

All studies must disclose on these points even when the disclosure is negative.

Sample size	No statistical method was used to predetermine sample size. Sample sizes were determined empirically according to our previous experiences and the review of similar experiments in literature.<br>Runyan et al., 2017 (PMID: 28723889), Makino et al., 2015 (PMID: 26167904), Steinecke et al., 2017 (PMID: 28584877)
Data exclusions	All data exclusions are described in the Methods section, and were predetermined. These included periods of immobility and cell ROI selection in the field of view.
Replication	We did not separately replicate our results with a new cohort of mice. However, in order to ensure reproducibility, we used both male and female animals, and we reported all the number of mice and neurons, all error bars, all p-values, and all r-values for all of our data. Our interpretation and conclusion are based on statistically significant results.
Randomization	Both male and female animals were randomly allocated to experimental groups. Different manipulation trials (Chemogenetic manipulation) of behavioral tasks were randomly determined. Bootstrapping procedures and tests were used for the calcium fluorescent dataset and confocal imaging dataset (see details in Method section).
Blinding	Data collection was not performed blind to the conditions of the experiments. However, we used the same behavioral protocols and custom-written codes for collecting and analyzing data.

Reporting for specific materials, systems and methods

We require information from authors about some types of materials, experimental systems and methods used in many studies. Here, indicate whether each material, system or method listed is relevant to your study. If you are not sure if a list item applies to your research, read the appropriate section before selecting a response.

Materials & experimental systems

n/a	Involved in the study
☐	☒ Antibodies
☒	☐ Eukaryotic cell lines
☒	☐ Palaeontology and archaeology
☐	☒ Animals and other organisms
☒	☐ Clinical data
☒	☐ Dual use research of concern

Methods

n/a	Involved in the study
☒	☐ ChIP-seq
☒	☐ Flow cytometry
☒	☐ MRI-based neuroimaging

Antibodies

Antibodies used	We used mouse anti-parvalbumin (supplier: Sigma) with 1:500 dilution, Cy5-conjugated donkey anti-mouse IgG (supplier: Jackson ImmunoResearch Laboratories) with 1:1,000 dilution, Rat anti-HA (supplier: Roche) with 1:500 dilution, Chicken anti GFP (supplier: ABCAM) with 1:800 dilution, and Mouse anti AnkyrinG (supplier: UC-Davis/NIH NEUROMAB) with 1:500 dilution.
Validation	All antibodies were validated by the companies we obtained them from prior to purchase. Additionally, the antibodies have been validated by multiple papers.<br>Steinecke et al., 2017 (PMID: 28584877), King et al., 2014 (PMID: 24477962), and Emilie Campanac et al., 2013 (PMID: 23439123)

Animals and other research organisms

Policy information about studies involving animals; ARRIVE guidelines recommended for reporting animal research, and Sex and Gender in Research

Laboratory animals	C57BL/6, PV-Cre (Cat.# 8069), SOM-Cre (Cat.# 13044) mice, Vipr2-Cre (Cat.#: 31332), Ai14 mice (Cat.# 7914) from Jackson laboratory (Bar Harbor, ME, USA) and Swiss Webster (SW, Cat.#24) mice from Charles River Laboratory (Boston, MA, USA) were used in this study (4-9 weeks old, both sexes). Nkx2.1-2a-CreER and ROSA-Flex-FlpO mouse lines were generated in the laboratory of Hiroki Taniguchi at the Max Planck Florida Institute for Neuroscience. All mice were maintained on a 12 hr light/ 12 hr dark cycle.
Wild animals	Our study did not involve wild animals.
Reporting on sex	Both male and female animals were used in this study and were randomly allocated to experimental groups. The number of male and females was about the same, 50% each.
Field-collected samples	Our study did not involve field-collected samples.
Ethics oversight	All experimental procedures were carried out in accordance with protocols approved by Johns Hopkins University Animal Care and Use Committee, the Max Planck Florida Institute for Neuroscience Institutional Animal Care and Use Committee, and National Institutes of Health guidelines.

Note that full information on the approval of the study protocol must also be provided in the manuscript.
